# Demonstration of flat-top beam illumination in widefield multiphoton microscopy

**DOI:** 10.1117/1.JBO.25.1.014503

**Published:** 2019-11-14

**Authors:** Mohammad M. Kabir, Hemangg S. Rajput, Varun A. Kelkar, Adriana C. Salazar Coariti, Kimani C. Toussaint

**Affiliations:** a University of Illinois at Urbana-Champaign, Department of Electrical and Computer Engineering, Urbana, Illinois, United States; b Laboratory for Photonics Research of Bio/Nano Environments (PROBE Lab), Urbana, Illinois and Providence, Rhode Island, United States; c University of Illinois at Urbana-Champaign, Department of Mechanical Science and Engineering, Urbana, Illinois, United States; d Brown University, School of Engineering, Providence, Rhode Island, United States

**Keywords:** flat-top beam, uniform illumination, multiphoton microscopy, two-photon fluorescence, second-harmonic generation

## Abstract

Multiphoton microscopy provides a suitable technique for imaging biological tissues with submicrometer resolution. Usually a Gaussian beam (GB) is used for illumination, leading to a reduced power efficiency in the multiphoton response and vignetting for a square-shaped imaging area. A flat-top beam (FTB) provides a uniform spatial intensity distribution that equalizes the probability of a multiphoton effect across the imaging area. We employ a customized widefield multiphoton microscope to compare the performance of a square-shaped FTB illumination with that based on using a GB, for both two-photon fluorescence (TPF) and second-harmonic generation (SHG) imaging. The variation in signal-to-noise ratio across TPF images of fluorescent dyes spans ∼5.6  dB for the GB and ∼1.2  dB for the FTB illumination, respectively. For the GB modality, TPF images of mouse colon and *Convallaria* root, and SHG images of chicken tendon and human breast biopsy tissue showcase ∼20% area that are not imaged due to either insufficient or lack of illumination. For quantitative analysis that depends on the illuminated area, this effect can potentially lead to inaccuracies. This work emphasizes the applicability of FTB illumination to multiphoton applications.

## 1 Introduction

Multiphoton microscopy (MPM) refers to an ensemble of imaging modalities that involve the simultaneous interaction of multiple photons with a material. MPM often requires longer illumination wavelengths (above 700 nm) compared to standard confocal microscopy, reducing optical absorption and scattering in biological tissues, thereby limiting tissue damage^1^ and increasing the penetration depth.^2^ Owing to its reduced photobleaching beyond the imaging planes,^3^ two- and three-photon fluorescence MPM modalities have been used for *in vivo* imaging of zebrafish pregnancy^4^ and observation of serotonin distribution in living cells,^5^ respectively. MPM techniques involving nonlinear scattering such as second-^6^^,^^7^ and third-harmonic generation^8^^,^^9^ (SHG and THG, respectively) take advantage of endogenous second- and third-order nonlinearities in biological systems leading to label-free imaging and tissue specificity. For example, SHG and THG microscopy have been used to differentiate healthy and injured horse tendon^10^ and study lymphocyte distribution in breast tissue biopsies, respectively.^9^

In a conventional multiphoton imaging system, the intensity distribution of the illumination beam is Gaussian. This results in only a segment at the center of the beam having sufficient intensity to generate a detectable multiphoton effect, whereas power in the edge of the beam is wasted. The beam power can be increased to ensure multiphoton interaction within a larger area of the beam, but this will mean that the signal-to-noise ratio (SNR) at various locations in the image would depend not only on the sample properties but also on the illumination intensity distribution.^11^ As such, the obtained image may not be a true representation of the sample’s structural or chemical composition, leading to erroneous results in any intensity-dependent quantitative analysis. In addition, increasing the beam power raises the possibility of photon-induced damage such as phototoxicity or bleaching of fluorophores, depending on the sample properties and MPM modality used. Interestingly, flat-top beams (FTB), i.e., beams with a uniform spatial intensity distribution, can help mitigate some of these aforementioned problems. Recently, FTBs have been employed for some linear microscopy techniques to obtain illumination field homogenization. In its simplest form, a rotating diffuser^12^^,^^13^ has been used to obtain a time-averaged speckle pattern of approximately uniform intensity distribution to improve the accuracy in single molecule localization. However, this method introduces moving components in the optical setup, leading to unwanted vibration in the imaging system. In addition, time-averaging constrains the imaging speed with respect to the speed of the spinning device. More importantly, the loss of coherence as light travels through the diffuser impedes the adoption of these techniques for coherent multiphoton processes such as SHG and THG. Another widely prevalent method for generating FTB involves the use of diffractive beam-shapers,^14^^–^^17^ which redistributes the illumination intensity by interfering various diffracted orders. These diffraction effects are accurate within a narrow spectral bandwidth, which limits the usage of these devices in multimodal MPM platforms that require multiple illumination wavelengths.^18^ Diffractive optics also create undesired diffraction orders, and their efficiency is strongly dependent on manufacturing tolerances. Alternatively, refractive phase elements have been used to redistribute the illumination GB to FTBs with ∼95% power throughput and ∼2.5% inhomogeneity in the intensity distribution. Recently, one such beam shaper has been used to increase the accuracy of single molecule localization for a super-resolution microscope.^11^ In this paper, we utilize a refractive Gaussian-to-top-hat beam shaper to generate a square-shaped FTB and compare its performance with that of a standard GB; both illumination types are investigated using a widefield MPM imaging system. Widefield imaging has been successfully demonstrated for two-dimensional (2-D) two-photon fluorescence (TPF) and SHG microscopy, albeit at the expense of requiring a higher average illumination power than that used in point-scanning techniques and no intrinsic axial sectioning capability.^19^^–^^22^ On this latter point, widefield illumination has either been combined with digital holography for three-dimensional (3-D) image reconstruction^23^^,^^24^ or temporal focusing^25^^–^^27^ to restrict the multiphoton effect to selected planes along the optical axis. In this paper, our analysis is performed in 2-D, which is sufficient for many practical applications that does not require 3-D reconstruction. For example, quantitative 2-D SHG and THG imaging has been used to assess the pathological conditions of breast tissue biopsies.^9^^,^^28^^–^^30^ In addition, 2-D imaging has also been shown to be useful in imaging of cultured neurons whose typical thickness are observed to be within a few tens of microns thereby limiting applicability of 3-D imaging.^20^ Furthermore, we characterize the effect of adopting a square-shaped uniform intensity distribution on the image contrast and on quantitation of features of interest obtained using TPF and SHG imaging modalities. We begin by simulating the effect of beam diameter and beam power on the intensity distribution of GBs and compare the beam power distribution with that of an FTB. Subsequently, we compare the 2-D spatial distribution of the SNR obtained from TPF images of a uniform layer of fluorescent dye using a widefield GB and a widefield FTB illumination mode. Next, we demonstrate the applicability of the FTB for biological tissue imaging by capturing TPF and SHG images of various biological tissues. We then demonstrate the effect of the reduced illuminated area caused by a GB illumination on the quantitative orientation analysis of obtained SHG images. The paper is organized as follows. Section 2 provides the methods; Sec. 3 provides the results and discussion. Finally, Sec. 4 provides the conclusion.

## 2 Methods

### 2.1 Sample Preparation

Fluorescein dyes, *Convallaria*, and mouse colon tissue were used for TPF, whereas chicken tendon and human breast tissue biopsy samples were used for SHG imaging in this study. Unstained *Convallaria majalis* (lily-of-the-valley) sample was purchased from MSMedia (New South Wales, Australia). The sample contained an array of two 1-mm sections of *Convallaria* tissue, which were fixed and mounted between a cover slip and a microscope slide. Each section has a thickness of 30  μm.

Mouse colon tissue was obtained locally from an abattoir and placed in tissue cassettes (Fisher Scientific). Next, it was processed in a standard xylene/ethanol mixture for 24 h for extensive dehydration. The sample was then embedded in paraffin wax using a tissue processor (Leica ASP300). Next, 8-μm-thick sections were cut using a cryostat (Leica CM3050S) and stained with hematoxylin and eosin (H&E) stain. Finally, each tissue section was mounted onto a microscope slide with a permanent mounting media (Permount).

Chicken tendon tissues were collected from a local abattoir and preserved in an embedding medium at −80°C. Next, the samples were slowly raised to a temperature of −20°C and 5-μm-thick sections were cut using a cryostat (Leica CM3050S). Subsequently, the samples were thawed and stained with hematoxylin and eosin (H&E) stain. Finally, the tissue section was mounted onto a microscope slide with a permanent mounting media (Permount).

Tissue microarray of breast tissue biopsy was purchased from US Biomax (Rockville, Maryland). The microarray consists of 1.5-mm-diameter cores, which are formalin-fixed and paraffin-embedded breast tissue samples. The samples include H&E-stained 5-μm-thick invasive ductal carcinoma tissue paired with normal breast tissue samples mounted on a microscope slide.

Two-photon polymerization (TPP) was performed with a photocurable polymer, which was prepared from a mixture of 98 ml poly(ethylene glycol) diacrylate (Sigma-Aldrich, CAS 26570-48-9), 2g Phenylbis (2,4,6-trimethylbenzoyl)phosphine oxide (Sigma-Aldrich, CAS 162881-26-7), and 0.02 g Sudan I (Sigma-Aldrich, CAS 842-07-9). To form a homogeneous mixture, all chemical components were mixed 24 h prior to usage and stored in a dark glass bottle to isolate the solution from ambient light. A drop of the mixture was placed on a microscope slide and covered with a cover slip to form a thin layer of the photopolymer.

### 2.2 Mathematical Model of the Flat-Top Beam

The expression for the optical field of an FTB with a square cross section can be modeled as^31^^–^^33^

$$U_{l}(x,y)=E_{0}g(\frac{x}{r_{i}},\frac{y}{r_{i}})\exp[i\beta \varphi (\frac{x}{r_{i}},\frac{y}{r_{i}})],$$ (1)

where $E_{0}$ is the amplitude, and $g(\frac{x}{r_{i}},\frac{y}{r_{i}})$ is the shape function of the incident beam, $\varphi (\frac{x}{r_{i}},\frac{y}{r_{i}})$ is the phase factor required to generate the FTB, and β is scaling factor expressed as

$$\beta =\frac{\pi \omega_{0}R_{o}}{f\lambda },$$ (2)

where $\omega_{0}$, $R_{o}$, f, and, λ refer to the diameter of the GB width of the FTB, focal length of the focusing lens, and wavelength of the beam, respectively. For an input GB, the field distribution can be described as

$$g(\xi )=\exp(-\frac{\xi^{2}}{2}),$$ (3)

where $\xi =(\frac{2\sqrt{2}x}{\omega_{0}},\frac{2\sqrt{2}y}{\omega_{0}})$, and $\omega_{0}$ is the diameter of the GB. The aforementioned phase factor φ can be expressed as

$$\varphi (\xi )=\frac{\pi }{2}\xi \;\mathrm{erf}(\xi )+\frac{1}{2}\;\exp(-\xi^{2})-\frac{1}{2},$$ (4)

where “erf” describes the error function. An ideal FTB is defined with an infinite intensity gradient at its edges, which is obtained when β→∞.

### 2.3 Experimental Setup

**Fig. 1:**
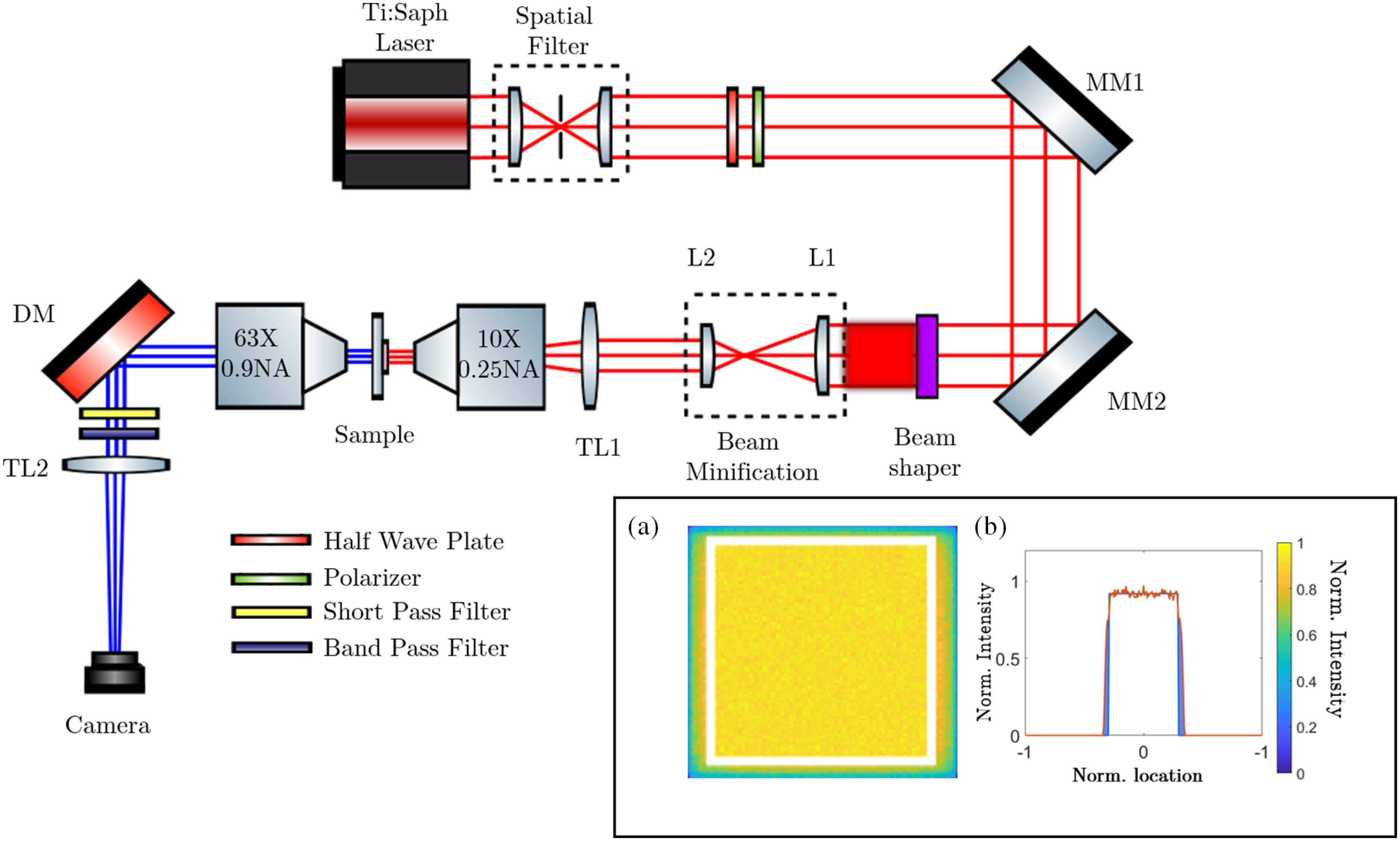
Schematic diagram of the experimental setup showing a modified MPM incorporating a square-shaped FTB illumination. Inset shows the simulated (a) 2-D and (b) 1-D intensity distributions of the FTB. MM1 and MM2, metal mirrors; L1 and L2, convex lens; TL1 and TL2, tube lens; DM, dichroic mirror.

Figure 1 shows the custom-built MPM setup used in this study. An ultrafast titanium:sapphire pulsed laser (Spectra-Physics Mai-Tai HP, Santa Clara, California) is used to generate a GB with 100-fs pulses at a repetition rate of 80 MHz. The laser is spectrally tunable between 690 and 1050 nm, and the excitation wavelength for this study is spectrally centered at 780 nm. An optical spatial filter is used to isolate the fundamental Gaussian mode and enlarge the beam diameter to 5 mm. A half-wave plate and a polarizer are used together to control power of the beam. Next, a pair of metallic mirrors reflects the beam onto the back aperture of a commercial Gaussian-to-top-hat beam shaper lens (TOPAG Lasertechnik GmbH, GTH-5-250-4-NIR, Darmstadt, Germany). A standard beam shaper comprises of a refractive phase-shaping element and a focusing element that redistributes a GB into a square-shaped FTB. In this case, a 5-mm-diameter GB is converted to an FTB of dimensions 4×4  mm at a distance of 250 mm. Due to the narrow 10-nm bandwidth of our 100-fs pulses and that the beam shaper is thin (4.0±0.1  mm), we do not observe any pulse broadening from using this optic. This observation is consistent with our analysis of the amount of group velocity dispersion experienced by our pulses upon passing through the beam shaper, which we calculate to be negligibly small at only ∼0.27%. Using relevant datasheets^34^^–^^36^ and formulae,^37^ step-by-step calculations were done to arrive at the result above, the specifics of which are provided in Supplementary Material 1. We use Zemax (OpticStudio, Kirkland, Washington) to simulate the propagation of the illumination beam through the beam shaper and visualize the resulting spatial intensity distribution of the FTB. To perform this simulation, a 3-D model of the beam shaper was obtained from the vendor (TOPAG Lasertechnik GmbH, Darmstadt, Germany). The inset in Figs. 1(a) and 1(b) shows the normalized 2-D and one-dimensional (1-D) intensity distribution of the FTB, respectively. We observe that the normalized root mean square value of the FTB is 0.92 for the flat region bounded by the white square shown in the 2-D intensity profile. Beyond this region, the intensity decreases with a sharp gradient (shown by shaded blue areas in the 1-D intensity profile).

After the generation of the FTB, a pair of convex lenses (f1=10  cm, f2=5  cm) are used to shrink the FTB to a size of 2×2  mm and relay the FTB on to the image plane of a tube lens TL1 (f3=40  cm). From the tube lens, the beam is passed onto a 0.25-numerical aperture (NA), 10× objective lens (Olympus America, Plan N FN22) that relays the FTB on to the sample plane. The size of the FTB at the sample plane is 60×60  μm. To obtain a GB illumination, the beam shaper is simply removed from the beam path, which leads to an illumination spot size of diameter 60  μm at the sample plane. Note that for both illumination conditions, a beam average power of ∼600  mW was used. Both forward propagating TPF and SHG signals are collected using a 0.9 NA condenser lens (Carl Zeiss Microscopy, Condsr Achr Apl 0.9 H D Ph DIC). The use of a lens with higher NA for collecting the emitted signal ensures that the spatial resolution of the imaging system is dictated by the NA of the collection objective.

The emitted signals are reflected by a dichroic mirror onto a laser-blocking filter (Semrock FF01-680/SP-25, Rochester, New York) and a relevant bandpass filter (Semrock FF01-530/40-25 for TPF, Semrock FF01-390/18-25 for SHG, Rochester, New York). Finally, the emitted signal is focused on to an EMCCD camera (Hamamatsu EM-CCD C9100-13, Bridgewater, New Jersey) with the help of tube lens TL2 (f4=30  cm). An image acquisition time of 1.5 s is utilized for all imaging modes.

## 3 Results and Discussion

**Fig. 2:**
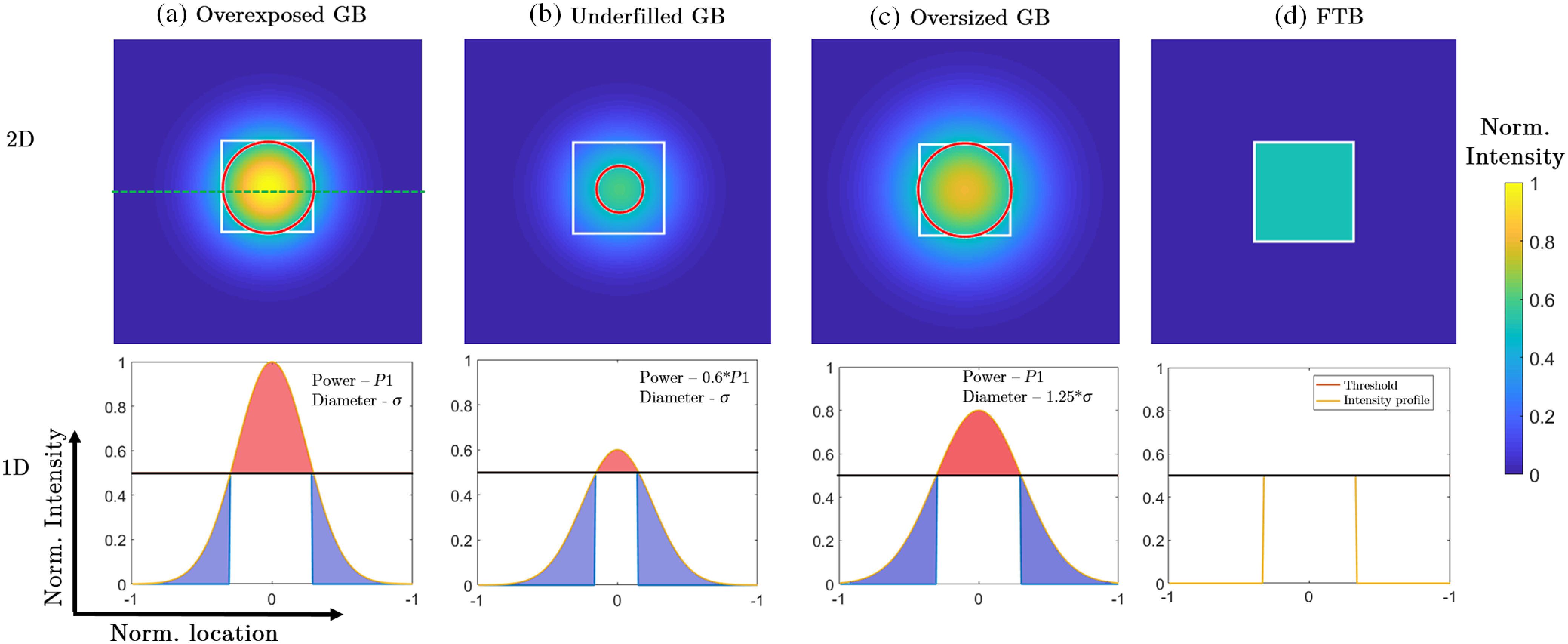
Comparison of the illumination intensity distribution of GB and FTB. Simulated 2-D transverse (x−y) intensity distributions are plotted for an (a) overexposed, (b) underfilled, and (c) overfilled GB, and compared with (d) an FTB. The white rectangle defines the imaging area and the red circles denote the diameter of the GB that corresponds to the intensity threshold. Dimensions for all 2-D plots are 2×2 arbitrary units. As shown by the green dashed line in (a), line intensity profiles are obtained along the horizontal direction and they are shown below the 2-D intensity plots. Overexposed regions are shaded in red, whereas underexposed regions are shaded in blue. See text for details.

We begin our study by comparing the theoretical power distribution for various cases of GBs and the FTB, the results of which are shown in Fig. 2. Using custom-written MATLAB (Mathworks, Natick, Massachusetts) code, we simulate the illumination intensity distribution for three different cases of the GBs over an arbitrary square area of 2×2 units. For all cases, a 2-D illumination intensity distribution is obtained, and the corresponding one-dimensional (1-D) line intensity profile calculated along the x-axis [horizontal green line in Fig. 2(a)] is shown below the 2-D plots. The intensity values are plotted on a normalized scale between 0 and 1. A value of 0.5 is chosen as an intensity detection threshold for two-photon processes (shown by a black line in the 1-D line intensity profiles), which is defined as the intensity value below which no detectable two-photon signal is obtained. This particular value was chosen to emphasize the difference in power distribution of the GB and FTB. For the purpose of this study, the threshold value is chosen arbitrarily, and it is not dependent upon the type of detector, sample, or two-photon process used. This threshold is kept constant for all illumination conditions with an aim to compare the effect of spatial intensity distribution on two-photon processes. For each beam, the power is obtained by calculating the area under the intensity profile. The white rectangle in Fig. 2 denotes the imaging area. As shown in columns (a) to (c), the three GBs are chosen based on beam diameter and power, whereby the red circle shows the size of the GB corresponding to the intensity threshold. For the first case of the GB shown in column (a), the diameter of the beam, defined by its full-width at half-maximum, is chosen to be 0.75 units, and the maximum intensity value is normalized to 1. For this GB, we observe that 51.9% of the beam power reaches the threshold (white rectangular area) and is optimally used for two-photon processes. We also note that 23.35% of the beam power is above the threshold (shown in red shaded area) leading to a significant beam overexposure to the sample. We also observe 24.75% of the beam power is below the two-photon intensity threshold (shown in blue shaded areas). With an aim to reduce the power distributed in overexposure, we consider a second case of the GB as shown in column (b), where the beam diameter is kept fixed, but the total power is reduced to 60% of its previous value. In this case, overexposure is reduced significantly to 2.53% of the total beam power, which is also depicted by the reduced red region in the 1-D line intensity profile. However, a larger portion of the beam amounting to 55.26% falls below the intensity threshold as indicated by the larger blue-shaded area and as such is not utilized for the two-photon process. Only 42.21% of the beam power reaches the two-photon threshold. For the final case of the GB shown in column (c), we consider an enlarged GB beam where the diameter is 1.25× larger than the GB considered in the first case. The total beam power is kept constant, which leads to a reduction of the maximum intensity by ∼25%. In this case, 18.45% of the beam power contributes to overexposure, whereas 32.85% of the beam power falls below the intensity threshold and does not contribute to the two-photon process. The portion of the beam power that is optimally utilized amounts to 48.7% for this case. Finally, column (d) shows the 2-D and 1-D intensity distribution for a square-shaped FTB that is obtained utilizing the mathematical formulation for the FTB depicted in Sec. 2. For an incident GB diameter of 5 mm, FTB width of 4 mm, incident wavelength of 780 nm, and focal length of the focusing lens of 250 mm, we evaluate the value of β to be 645 as defined by Eq. (4) of Sec. 2. For the purpose of comparison, the width of the FTB is chosen to be equal to the diameter of the GB in the first case (0.75 units), and its intensity is chosen to be equal to the two-photon intensity threshold. From the 2-D and 1-D intensity profiles shown in Fig. 2(c), we observe that a negligible part of the beam is underutilized or distributed in overexposure. The corresponding values for overexposure, underexposure, and optimum utilization are 1.77%, 9.14%, and, 89.09%, respectively, for the square-shaped FTB obtained by simulating the beam propagation through the beam shaper. From the results of this study summarized in Table 1, it is obvious that a uniform intensity distribution leads to a significant increase in the utilization of the beam power for two-photon processes.

**Table 1:** Comparison of the power distribution in GBs and FTBs.

	Overexposed GB	Underfilled GB	Oversized GB	Theoretical FTB
% Overexposed	23.35	2.53	14.37	1.77
% Underexposed	24.75	55.26	37.64	9.14
% Optimally utilized	51.9	42.21	48.7	89.09

**Fig. 3:**
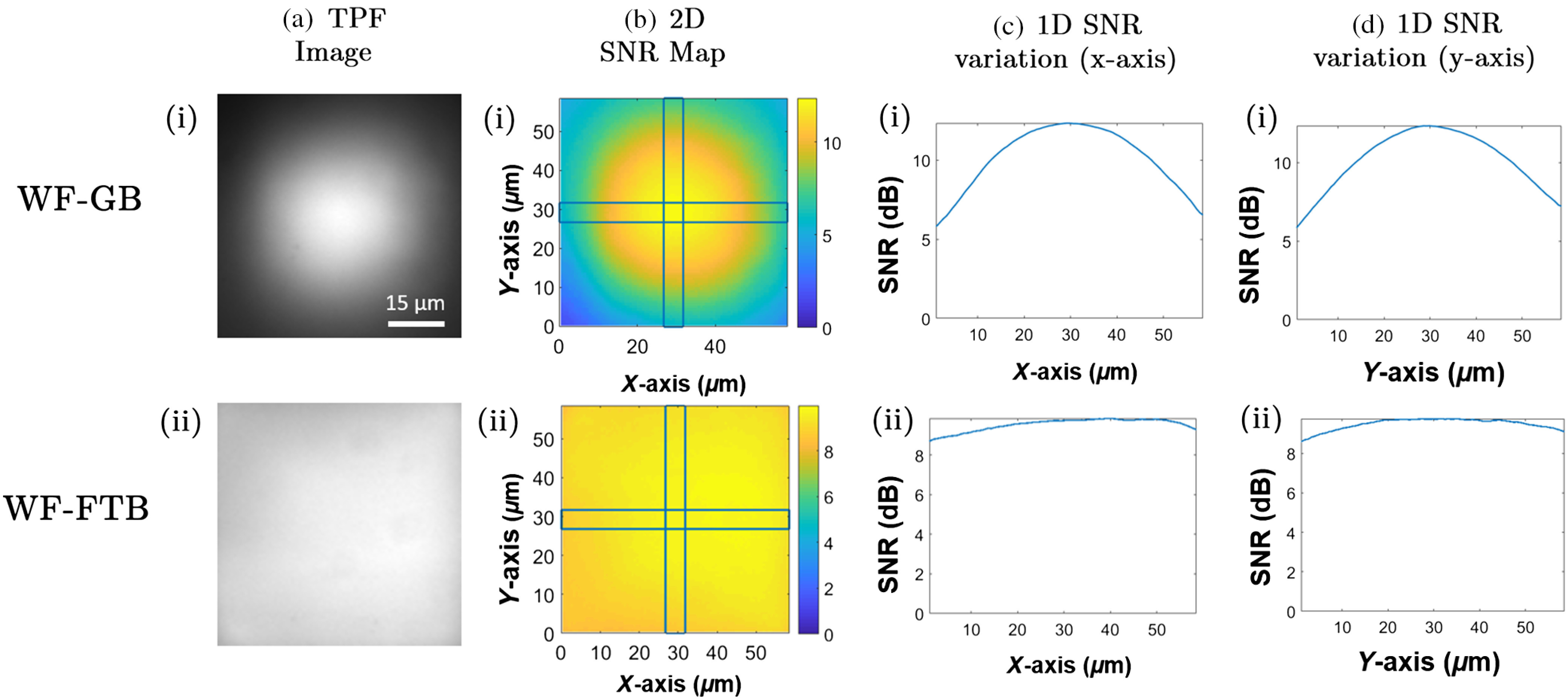
Comparison of the SNR distribution in TPF images. (i) GB and (ii) FTB illumination are used to obtain (a) TPF images of a uniform layer of fluorescent dye. For each case, the corresponding (b) 2-D and 1-D SNR distribution along the (c) x- and (d) y-axes is demonstrated.

Next, we compare the distribution of SNR on TPF images of a uniform layer of fluorescent dyes using a widefield GB and a widefield FTB illumination systems, the results of which are shown in Figs. 3(i) and 3(ii)), respectively. Column (a) depicts the TPF images, where we clearly notice the disparity in intensity distribution between these two beams. From these images, we calculate the SNR for each point within the image and develop a 2-D SNR map as shown in column (b). The noise value in the SNR map corresponds to the constant thermal and dark noise in the detector, and it is obtained by calculating the average intensity of a 25×25pixel region in an image captured by the camera without the presence of the illumination beam. From the 2-D TPF SNR map for the GB shown in (b, i), we observe a significant variation in SNR between the periphery and the center of the image, whereas the corresponding 2-D TPF SNR map for FTB shown in (b, ii) demonstrates only a modest variation in its SNR distribution. These results are further clarified by observing the 1-D SNR variation along the x- and y-axes shown in columns (c) and (d), respectively. To obtain the 1-D SNR distribution along the x-axis, we define a horizontal rectangular region of 5×60  μm for both 2-D SNR maps as shown in column (b) and plot the average SNR values within that region. A similar procedure is adopted for the 1-D SNR distribution along the y-axis, whereby the average SNR values obtained from a 60×5  μm vertical rectangle are plotted. As shown in (c,i) and (d,i), the SNR variation between the edge and center of the image for GB is 6.59 and 6.62 dBs along the x- and y-axes, respectively. The corresponding variation for the FTB shown in (c,ii) and (d,ii) is 1.32 dBs for both the x- and y-axes, respectively. Note that similar to TPF, TPP^38^^–^^40^ can also be used to compare the intensity distribution of an FTB and a GB. As such, we perform TPP with an FTB illumination and compare the results with a GB illumination, the results of which are discussed in Supplementary Material 2.

**Fig. 4:**
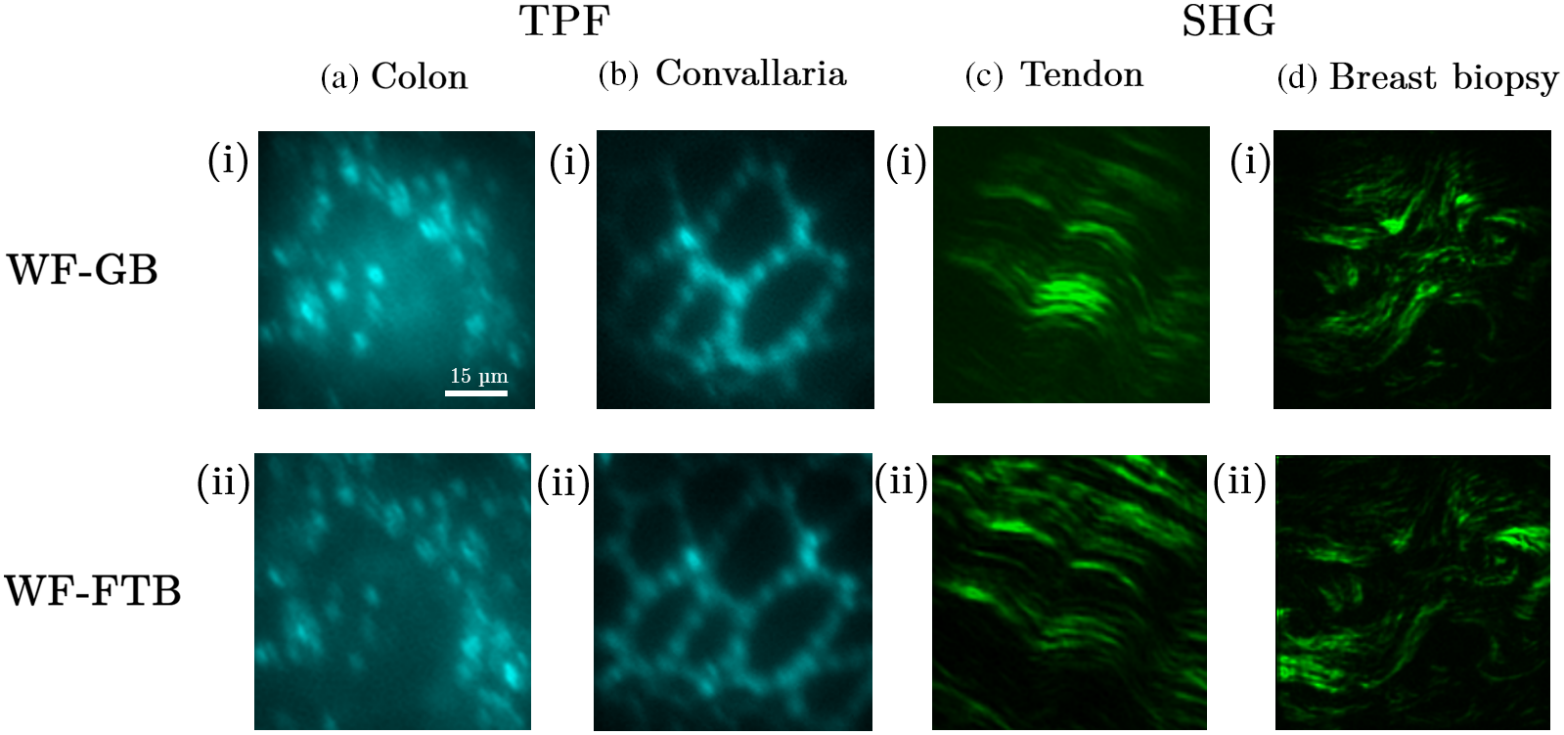
Application of FTB in MPM imaging of biological tissues. TPF images of (a) colon and (b) *Convallaria* roots and SHG images of (c) chicken tendon and (d) human breast biopsy are obtained using (i) GB and (ii) FTB imaging platforms.

Next, we apply the FTB imaging platform to obtain TPF and SHG images of biological tissues and compare the illumination of the region of interest in the imaging plane with a GB imaging modality. Four different tissue types were chosen showing biological structures of varying shapes and sizes. As shown in Fig. 4, mouse colon and *Convallaria* rhizome tissue are chosen for TPF imaging, whereas chicken tendon and human breast biopsy tissue are chosen for SHG imaging. The TPF images of the colon shown in Fig. 4(a) showcase a collection of immune cells, whereas Fig. 4(b) shows a cross-section of the *Convallaria* rhizome. In both cases, GB shows lack of illumination in the corners of the image, which results in several immune cells in the colon and some portion of the *Convallaria* vascular boundaries appearing with a poor contrast or not appearing at all. We also note a strong background signal in Fig. 4(a,i), which is significantly reduced in Fig. 4(a,ii). As the GB is overexposed toward the center, a stronger out-of-plane signal is detected in the GB modality, which contributes to the stronger background signal. Figures 4(c) and 4(d) show similar effects of reduced illumination in SHG images of tendon and breast biopsy. Tendon has thicker, densely packed, uniformly oriented collection of collagen fibers, whereas the fibers in the breast tissue are comparatively more fragmented, sparsely distributed, and randomly oriented. Despite these differences, both sets of images show observable lack of illumination at the corners and overexposed contrast near the center of the image in images taken with GB modality. By comparing 10 sets of images taken with GB and FTB illumination for each type of tissue, we observe that the images obtained with GB showcases ∼20% more dark area than images taken with FTB.

**Fig. 5:**
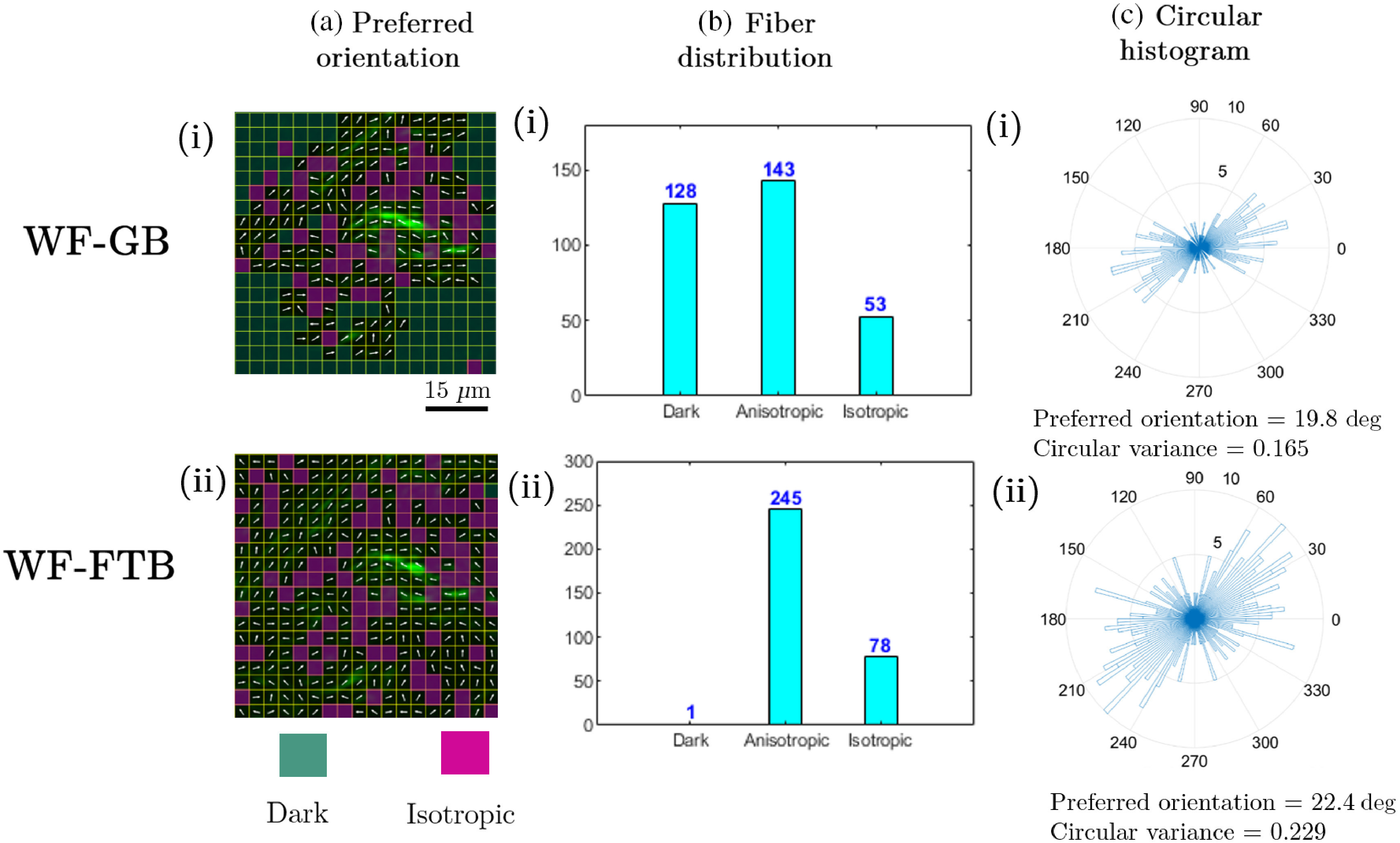
Effect of illumination distribution on orientation analysis of SHG images. FT-SHG analysis was applied to obtain (a) preferred orientation of collagen fibers in SHG images of breast biopsy tissue obtained with (i) GB and (ii) FTB illumination. (b) The bar plot demonstrates the number of dark, anisotropic and isotropic regions, whereas a (c) circular histogram shows the distribution of fiber orientation within the anisotropic regions.

The aforementioned observations regarding the use of GB illumination can affect the accuracy of quantitative analysis for metrics that are dependent on the area of the illuminated region. More specifically, any feature located near the corners of an image will not be included in the analysis, which can lead to erroneous evaluation of the structural content of the whole image. To elucidate this factor, we applied 2-D Fourier transform-based orientation analysis (FT-SHG) on SHG images of breast biopsy tissue, the results of which are shown in Fig. 5. The details of this analysis technique are presented elsewhere.^7^ Briefly, we divide the images into 18×18 grids, where each grid has a dimension of 8×8  pixels. For an image dimension of 60×60  μm, this results in 324 grids. If the average intensity within each grid is below a predefined noise threshold, the grid is labeled as a dark region and overlaid with a cyan color. Next, the variation in fiber orientation within each grid is used to define it as an anisotropic or an isotropic region; it is overlaid with a magenta color in the latter case. Finally, the preferred orientation of fibers within each anisotropic grid is calculated and displayed. By comparing the results of this analysis, we observe from Fig. 5(a) that the image taken with GB illumination exhibits significantly more dark regions compared to the image taken with FTB illumination. From the bar plot summarizing the number of dark, anisotropic and isotropic regions shown in Fig. 5(b), we observe that 127 more grids are marked as dark in the image captured with GB, which reduces the number of isotropic and anisotropic regions by 25 and 102, respectively. These discrepancies lead to differences in the orientation analysis, which is depicted in the circular histograms shown in Fig. 5(c). We observe that the preferred orientation of fibers is 19.8 deg for the SHG image taken with GB, whereas the value is 22.4 deg for the image obtained with FTB. The distribution of fiber orientation between anisotropic grids is depicted by the circular variance, which assumes values of 0.165 and 0.229 for images taken with GB and FTB imaging modalities, respectively. We attribute this discrepancy to the fact that ∼20% more area is considered for orientation analysis in the FTB image.

Notwithstanding the applicability of FTB illumination for multiphoton imaging and manufacturing applications, it does come with some limitations. Due to the use of a low NA objective lens to create the widefield illumination, the axial extent of the beam is on the order of tens of microns. This factor leads to a reduced axial resolution and hampers the formation of 3-D images. In addition, increasing the illumination area leads to increased speckle for coherent optical processes such as SHG. In the current implementation of FTB, speckle formation is observed for tissue samples thicker than 30  μm. This sample thickness is consistent with those used in previously reported MPM modalities.^26^^,^^41^^–^^43^ In light of these limitations, we have focused our work on 2-D analysis, which is sufficient for many practical applications and does not require 3-D reconstruction. For example, it is a standard practice among pathologists to rely on 2-D images obtained from thin tissue samples for pathological studies. Moreover, quantitative 2-D SHG imaging^28^^–^^30^ and 2-D THG imaging^9^ have previously been applied to assess the pathological conditions of breast tissue biopsies. In addition, 2-D imaging alone has also been shown to be useful in imaging of cultured neurons, which is typically within a few tens of micrometers.^20^ Thus, our findings could readily find utility in similar aforementioned cases, where single-shot multiphoton imaging of thin structures at the micron scale is attractive, and transverse variations in sample morphology in 2-D can be obtained without bias to the illumination beam’s intensity distribution.

## 4 Conclusion

In summary, we utilized a square-shaped FTB illumination to perform multiphoton microscopy under widefield illumination conditions. Compared to a GB illumination, the use of an FTB provides ∼40% more optimal utilization of beam power in two-photon imaging applications. From TPF imaging of fluorescent dye, we observed a ∼5.6 and 1.2 dB difference in SNR variation between the edge and the center of the beam for GB and FTB illumination profiles, respectively. Moreover, we found ∼20% more dark areas in TPF images of mouse colon and *Convallaria*, and SHG images of chicken tendon and human breast biopsy tissue obtained with a GB illumination. The potential effect of reduced illuminated area in quantitative orientation analysis of collagen fibers was demonstrated by a ∼12% difference in fiber orientation and ∼7% difference in circular variance in SHG images of breast biopsy tissue when using GB illumination. Our work highlights the various potential benefits from considering FTB as an illumination modality for multiphoton applications.
