# Supplementary material for: Demonstration of flat-top beam illumination in widefield multiphoton microscopy
Source: J Biomed Opt. 2019 Nov 14;25(1):014503. doi: 10.1117/1.JBO.25.1.014503 (PMC7008505; doi:10.1117/1.JBO.25.1.014503)
Supplement: Supplementary file 1 [file JBO_025_014503_SD001.pdf]

Supplemental 1
Demonstration of flat-top beam illumination in widefield multiphoton microscopy

MOHAMMAD M. KABIR,[1,2] HEMANGG S. RAJPUT,[2,3] VARUN A. KELKAR,[1,2] ADRIANA C. SALAZAR COARITI,[2] AND KIMANI C. TOUSSAINT, JR.[2,3,4*]

[1]University of Illinois at Urbana-Champaign, Department of Electrical and Computer Engineering, Urbana, IL, USA
[2]University of Illinois at Urbana-Champaign, Photonics Research of Bio/Nano Environments, Urbana, IL, USA
[3]University of Illinois at Urbana-Champaign, Department of Mechanical Science and Engineering, Urbana, IL, USA
[4]Brown University, School of Engineering, Providence, RI, USA

Calculation of maximum pulse broadening due to group velocity dispersion in flat top beam converter

- We first calculate the refractive index for the material of the beam shaper (LF5 Schott glass, as per the beam converter datasheet[34]) at the center wavelength $\lambda_0 = 780$ nm, as well as for $\lambda_1 = 775$ nm and $\lambda_2 = 785$ nm, corresponding to an effective bandwidth of 10 nm around the center wavelength[35]:

$$n^2(\lambda) = 1 + \frac{B_1\lambda^2}{\lambda^2 - C_1} + \frac{B_2\lambda^2}{\lambda^2 - C_2} + \frac{B_3\lambda^2}{\lambda^2 - C_3} \quad (1)$$

Here, n is the refractive index, λ is the wavelength in microns, and $B_{1,2,3}$ and $C_{1,2,3}$ are the Sellmeier coefficients provided in the datasheet of LF5 Schott glass[36]:

$B_1 = 1.28035628$

$B_2 = 0.163505973$

$B_3 = 0.893930112$

$C_1 = 0.00929854416$

$C_2 = 0.0449135769$

$C_3 = 110.493685$

Based on the refractive index values for these three wavelengths, $\frac{d^2n}{d\lambda^2}$ at 780 nm is calculated using the finite difference approximation

$$\frac{d^2n}{d\lambda^2} = \frac{n(\lambda_0+5\ nm)+n(\lambda_0-5\ nm)-2n(\lambda_0)}{(5\ nm)^2} \quad (2)$$

- Now, we need to only consider those spatial frequencies that are allowed by the NA of the illumination objective. The highest spatial frequency allowed will have the longest (slant) path L through the beam shaper. We approximately calculate that based on the thickness of the beam shaper and the angle the highest spatial frequency ray makes with the optic axis:

$$L = \frac{L_0}{cos(sin^{-1}(\theta))} \quad (3)$$

Then, we calculate the group velocity dispersion related term using equation 4.21[37]:

$$\psi_2 = -\beta_2 L = \frac{-\lambda^3\ L}{2\pi c^2}\ \frac{d^2\ n}{d\lambda^2} \quad (4)$$

- From this, and using the input pulse width of 100 fs (10^{-7} us), we calculate the output pulse width using the following formula from equation 4.25[37]:

$$\frac{\Delta\tau_{out}}{\Delta\tau_{in}} = \sqrt{1 + \frac{4\psi_2^2}{\Delta\tau_{in}^4}} \quad (5)$$

- From the resulting calculations we see that the pulse broadening is around 0.27%, which can be assumed to be negligible.
