# Supplementary material for: Demonstration of flat-top beam illumination in widefield multiphoton microscopy
Source: J Biomed Opt. 2019 Nov 14;25(1):014503. doi: 10.1117/1.JBO.25.1.014503 (PMC7008505; doi:10.1117/1.JBO.25.1.014503)
Supplement: Supplementary file 2 [file JBO_025_014503_SD002.pdf]

Supplemental 2
Demonstration of flat-top beam illumination in widefield multiphoton microscopy

MOHAMMAD M. KABIR,[1,2] HEMANGG S. RAJPUT,[2,3] VARUN A. KELKAR,[1,2] ADRIANA C. SALAZAR COARITI,[2] AND KIMANI C. TOUSSAINT, JR.[2,3,4*]

[1]University of Illinois at Urbana-Champaign, Department of Electrical and Computer Engineering, Urbana, IL, USA
[2]University of Illinois at Urbana-Champaign, Photonics Research of Bio/Nano Environments, Urbana, IL, USA
[3]University of Illinois at Urbana-Champaign, Department of Mechanical Science and Engineering, Urbana, IL, USA
[4]Brown University, School of Engineering, Providence, RI, USA

Two photon polymerization

In two-photon polymerization (TPP), two-photon absorption in the resin leads to cross-linking and polymerization, which results in fabrication of the structure. TPP performs maskless (direct laser) lithography that enables fabrication of sub-micron scale structures for applications in optical communication[38], biological sensing[39] and MEMS devices[39,40]. Since the fidelity of the structure manufactured depends on the two-photon absorption process, TPP is fundamentally governed and limited by very similar principles as TPF microscopy. Hence, we expect that using a FTB in TPP would lead to similar advantages, such as simultaneous polymerization of all of the desired manufacturing area in a photosensitive resin and a uniform polymerization rate across all of the illuminated region. These two factors would potentially ensure minimal undesired variations in the structures manufactured. To demonstrate this advantage, we perform TPP with a FTB and compare the results with a GB illumination, the results of which are shown in Fig. S1. The illumination configuration shown in the experimental setup in Fig. 1 was adapted for this process. For both illumination conditions, the photosensitive resin is exposed to a beam of ~580 mW average power for a duration of 30 seconds, to ensure the average intensity is sufficient for complete polymerization. Using a GB illumination, we observe that only the central area is polymerized, where the intensity of light is higher than the polymerization threshold of the resin. As the intensity

decreases from the center towards the edge, the manufactured structure loses its integrity and becomes ill-formed. In contrast, we observe a comparatively uniform structure with sharply defined boundaries when FTB was used for the manufacturing process. This observation suggests uniform and complete polymerization throughout the entire illuminated area.

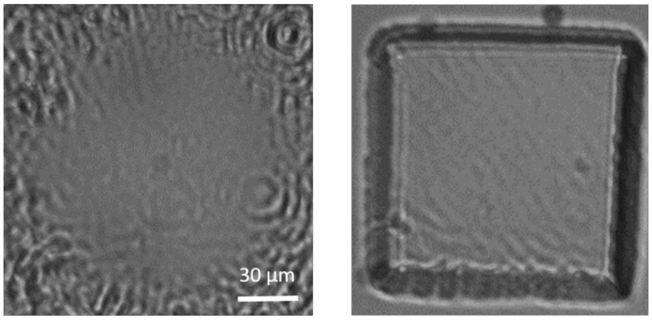

Fig. S1: Two-photon polymerization using (a) WF-GB, and (b) WF-FTB illumination.
